# Development of a Candidate Multi-Epitope Subunit Vaccine against *Klebsiella aerogenes*: Subtractive Proteomics and Immuno-Informatics Approach

**DOI:** 10.3390/vaccines9111373

**Published:** 2021-11-22

**Authors:** Ahitsham Umar, Asma Haque, Youssef Saeed Alghamdi, Mutaib M Mashraqi, Abdur Rehman, Farah Shahid, Mohsin Khurshid, Usman Ali Ashfaq

**Affiliations:** 1 Department of Bioinformatics and Biotechnology, Government College University Faisalabad, Faisalabad 38000, Pakistan; ahitshamumar85@gmail.com (A.U.); asma@gcuf.edu.pk (A.H.); abdurrehman93@gcuf.edu.pk (A.R.); farahshahid24@gcuf.edu.pk (F.S.); 2 Department of Biology, Turabah University College, Taif University, Taif 21944, Saudi Arabia; ysghamdi@tu.edu.sa; 3 Department of Clinical Laboratory Sciences, College of Applied Medical Science, Najran University, Najran 61441, Saudi Arabia; mmmashraqi@nu.edu.sa; 4 Department of Microbiology, Government College University Faisalabad, Faisalabad 38000, Pakistan; mohsinkhurshid@gcuf.edu.pk

**Keywords:** subtractive proteomics, subunit vaccine, *Klebsiella aerogenes*, molecular docking

## Abstract

*Klebsiella aerogenes* is a Gram-negative bacterium which has gained considerable importance in recent years. It is involved in 10% of nosocomial and community-acquired urinary tract infections and 12% of hospital-acquired pneumonia. This organism has an intrinsic ability to produce inducible chromosomal AmpC beta-lactamases, which confer high resistance. The drug resistance in *K. aerogenes* has been reported in China, Israel, Poland, Italy and the United States, with a high mortality rate (~50%). This study aims to combine immunological approaches with molecular docking approaches for three highly antigenic proteins to design vaccines against *K. aerogenes*. The synthesis of the B-cell, T-cell (CTL and HTL) and IFN-γ epitopes of the targeted proteins was performed and most conserved epitopes were chosen for future research studies. The vaccine was predicted by connecting the respective epitopes, i.e., B cells, CTL and HTL with KK, AAY and GPGPG linkers and all these were connected with N-terminal adjuvants with EAAAK linker. The humoral response of the constructed vaccine was measured through IFN-γ and B-cell epitopes. Before being used as vaccine candidate, all identified B-cell, HTL and CTL epitopes were tested for antigenicity, allergenicity and toxicity to check the safety profiles of our vaccine. To find out the compatibility of constructed vaccine with receptors, MHC-I, followed by MHC-II and TLR4 receptors, was docked with the vaccine. Lastly, in order to precisely certify the proper expression and integrity of our construct, in silico cloning was carried out. Further studies are needed to confirm the safety features and immunogenicity of the vaccine.

## 1. Introduction

Klebsiella aerogenes is a rod-shaped Gram-negative motile bacterium that belongs to the Enterobacteriaceae family. According to Hormaeche and Edwards, in 1960, it was formerly named Enterobacter aerogenes [1,2]. K. aerogenes is a pathogenic bacterium that is commonly associated with nosocomial outbreaks and has been associated with numerous infections involving bloodstream infections, skin and tissue infections, respiratory infections and urinary tract infections [3]. The multidrug resistant (MDR) strains of K. aerogenes that are emerging are responsible for higher death rates in ICU patients [3]. K. aerogenes has become a major opportunistic pathogen with epidemic potential. Hypoalbuminemia, invasive procedures such as inserting a drainage tube or a urinary catheter and past carbapenem exposure have all been linked to cabapenem-resistant K. aerogenes (CRKA) infections.

*K.aerogenes*, as well as *K. pneumoniae*, *Enterobacter cloacae* and *E. coli*, has been identified as one of the most commonly isolated carbapenem-resistant *Enterobacteriaceae* (CRE) in the USA and other regions of the world [4,5,6]. In the United States and around the world, clinical CRKA strains with plasmid-borne serine carbapenemases were documented, while Mettallo-beta-lactamases and OXA-48 were discovered in Europe, Asia and Brazil [3,7]. According to previous research works, Carbapenem resistance in *K. aerogenes* is thought to be caused by overexpression of ESBLs or ampC enzymes, as well as mutations that impair membrane permeability [3]. In different countries, carbapenemases, such as KPC, NDM and OXA-48, were reported from the clinical isolates of *K. aerogenes* [8].

Despite the fact that genetic diversity and specific sequence types (STs) are significant in *K. aerogenes*, the genomic data have shown that *K. pneumoniae*, *E. cloacae* and *K. aerogenes* are related to each other, when geographical spread, multiple drug resistance, hospital outbreaks and disease syndromes were reported [9,10]. A multilocus sequence typing (MLST) structure for *K. aerogenes* has recently been developed, but its performance has not been made publicly available.

Public health is at serious risk due to bacterial infections that may lead to hazardous infections, failure of organs, decomposition and, occasionally, death. From all over the world, around 10 million people are infected through bacterial infections. Healthy bacteria and tissues are destroyed by foreign bacteria when they enter the body and spread their population, which causes infections. *K. aerogenes* is one of the very lethal pathogens that may cause skin, tissues, respiratory and urinary tract infections in humans. A high rate of mortality is found in ICU patients due to *K. aerogenes*. This pathogen is highly resistant against drugs and antibiotics that are used for its treatment. Therefore, it is direly needed to develop drugs or vaccines against this pathogen [11]. Antibiotics have proved to be highly effective in treating a number of human infections and a preventative method for a variety of clinical treatments [12]. Antibiotic resistance is accelerated by the extensive and frequently ineffective use of antibiotics [13,14]. *K. aerogenes* isolates that harbor an extended range of beta-lactamase and carbapenemase genes and such strains are frequently associated with hospital outbreaks [15]. Recent research suggests that carbapenem resistance in *K. aerogenes* is mostly caused by overexpression of ESBLs and ampC enzymes harbored by efflux pumps and efflux pump inhibitors (EPIs) [16].

Antigenic protein fragments that can duplicate the actions of the current pathogen and elicit an immune response to the pathogen might be supported by vaccine components [10]. Antigenic protein fragments in the vaccine’s subunits can copy the real pathogen and activate an immune response against the targeted bacteria [17,18,19,20,21,22,23,24]. We anticipate that the findings of this report will construct a new candidate vaccine for *K. aerogenes.*

## 2. Methodology

**Figure 1:**
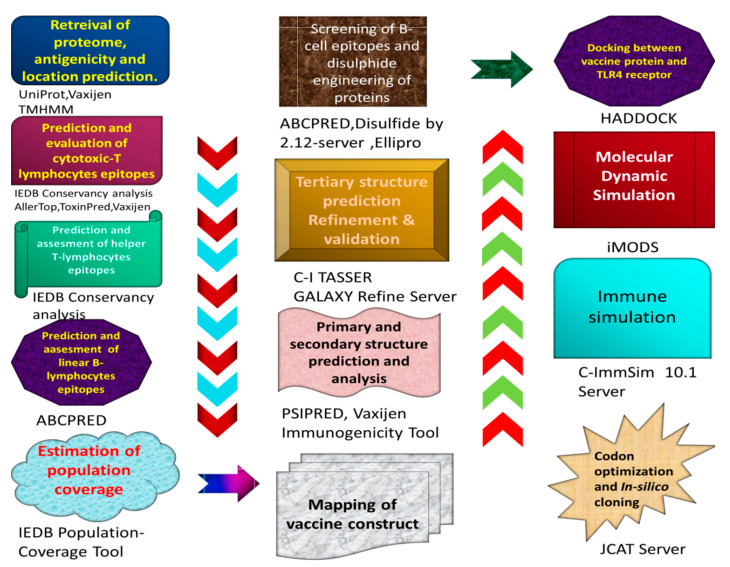
The immuno-informatics approaches utilized to create MEBSV are depicted in this diagram.

Flow diagram of the entire procedure selected in this research work is demonstrated in Figure 1.

### 2.1. Retrieval of Proteom Analysis

UniProt was used to extract the entire proteome of the *K. aerogenes* strain in FASTA format [25]. The essential proteins were found using the Geptop 2.0 server. To avoid an autoimmune response, vaccine candidates should not be homologous to human proteins. BLASTp was used to predict non-homologous proteins [26]. PSORTb 3.0.3 was used for cellular localization, to select the outer membrane proteins. For peptide-based vaccine designing, a protein was selected with higher antigenicity, because antigenicity refers to a person’s ability to respond rapidly and be immune to an antigen. The Vaxijen 2.0 server was utilized to evaluate the antigenicity of all proteins of *K. aerogenes* with a threshold of 0.5 [27]. When using the autocross covariance transformation procedure, this server has an accuracy of 70–89 percent [27]. In target protein selection, proteins with transmembrane helices were not required due to their problematic nature during purification. The TMHMM v-2.0 server was used to predict the transmembrane helix [28].

### 2.2. CTL Epitope Selection and Evaluation

Cytotoxic T cells play an essential part in selecting particular antigens for the perfect design of CTL epitopes that are important for the vaccine. A tool called (http://tools.immuneepitope.org/mhci, accessed on 20 November 2021) was used for the prediction of MHC-I binding, by which 40 MHC-I epitopes were predicted. For the prediction of CTL epitopes, a consensus method was applied after the submission of protein sequence in FASTA format; species from humans were selected as a source and all cast epitopes were chosen. The epitopes were chosen when there was a score of consensus < 2, due to the strong ability of binding [29].

For immunogenicity of the CTL epitopes, the immunogenicity tool was used via online resources (IEDB MHC-I) [29]. To check if the epitopes could produce a reflex in the immune response, the Vaxijen-v-2.0 server was used to calculate the antigenicity. Reactions that are allergic or poisonous should be avoided, during the construction of the vaccine; therefore, an online server, Aller-TOP 2.0, was utilized to calculate the allergenicity of epitopes. The Toxin-Pred server was used for the identification of non-toxic CTL epitopes [30].

### 2.3. Selection and Analysis of HTL Epitopes

Helper T cells, so-called CD4-positive T lymphocytes, have a critical role in adaptive immunity for the humoral and cellular immune responses to foreign antigens. That is why HTL epitopes connected with MHC (Major histocompatibility complex) -II alleles are very important for vaccine designing [31]. To promote B cells to produce antibodies against effective pathogens’ cells in response to cytotoxic T cells and pathogens, T cells play a very elementary role [32]. The 27 HTL epitopes were calculated by a selected percentile threshold of 2, while keeping the consensus method using the IEDB’s (Immune Epitope Database) MHC-II binding tool on the chosen proteins [33]. Different cytokines were generated, such as IFN-gamma, IL-4 and IL-10, leading to the recruitment of various CTL and other cell-mediated immune responses. In the construction of a vaccine, cytokine-inducing HTL epitopes are essential. The IFN epitope server was utilized selecting hybrid (SVM and motif) methods while keeping the remaining parameters, such as IFN versus non-IFN model, by default [34]. For the comparison of the inducing properties of IL-4 and IL-4Pred [35], as well as for IL-10, IL-10Pred was used [36]. In IL4Pred and IL10Pred, SVM threshold values of 0.2 and −0.3 were used, respectively. The activation of cellular and humoral immune responses is aided by helper T lymphocyte (HTL) responses. As a result, HTL epitopes are anticipated to have a leading role in preventive and therapeutic vaccinations.

### 2.4. LBL Epitope Identification and Evaluation

B-cell epitopes are essential building blocks of a vaccine, due to their importance in the immune system to enhance the adaptive immune response [37]. B-cell epitopes were predicted through ABCPred [38]. The minimum value for forecasting was set to be 0.5 for linear epitopes. Online servers such as VaxiJen v-2.0 and AllergenFP, along with ToxinPred, were used for profiling antigenicity and allergenicity, followed by toxicity [30,31,39]. A vaccine should have conserved epitopes and can elicit specific B-cell and T-cell (CD4 and CD8) responses. By including highly conserved B-cell epitopes in the construct, pathogen-specific immune responses can be stimulated with negligible side effects [39].

### 2.5. Vaccine’s Mapping

An adjuvant and linkers combined with the proposed epitopes result in the vaccine construct [40,41]. To increase the immunogenicity of the construct, the adjuvant is used very carefully. If the selection of peptides is performed on its own, then these could be poorly immunogenic [42]. Cholera enterotoxin subunit B (accession No.: P01556) was utilized as an adjuvant [43]. The EAAAK linkers were utilized to combine the first CTL epitope and adjuvant and to isolate the domains of a bi-functional fusion protein. For effective epitope functionality, a linker was used to bind two epitopes [44]. The AAY linker was combined with CTL, while GPGPG was linked with HTL for the successful construction of the vaccine construct and also for the identification of epitopes. The KK linker was combined with LBL epitopes to form a fusion peptide.

### 2.6. Structural Analysis

For the confirmation of the MEBSV sequence, designed as non-homologous, first, the Blastp analysis was performed [45]. The following physiochemical properties were evaluated through the ProtParam server: pI = isoelectric point and MW = molecular weight; these were followed by GRAVY = grand average of hydropathicity and in vivo, along with in vitro, half-life [45,46]. The Vaxijen-v-2.0 server and IEDB immunogenicity tools were used to evaluate the antigenic and immunogenic profiles. The AllerTOP server was used to analyze the vaccine’s allergenic reactions. The secondary structure of the vaccine construct was determined by employing the PSIPRED workbench. The primary purpose behind this software usage is that it estimates other qualities, such as α-helices, degree of β-turns, random-coil and extended-chain [47].

### 2.7. Prediction of Tertiary Structure, Confirmation and Refinement

A protein’s 3D structure is its lower energy structure, which twists and bends precisely to validate the highest stability. The development of our protein was performed through the I-TASSER server, which used connection information to enhance the structural accuracy of the protein [48]. The refinement and optimization of the 3D-structured protein was accomplished through the Galaxy Refine server [49]. In the dynamic simulation, the general systemic relaxation ruined the structure. Our refined structure was validated by employing the RAMPAGE server, which utilizes the Ramachandran plot [50]. In further stages, the ProSA-web server was used for structural verifications. In the vaccine structure, the data review on non-bonded relations was performed through the ERRAT server [51].

### 2.8. B-Cell Epitopes Screening

Linear/conformational B-cell epitopes were found out by utilizing an online server such as IEDB-AR v.2.22. For the ABCPred server, the vaccine sequence was used as an input with a set value of 14 and a threshold of 0. The vaccine structure was given as input and all Ellipro tool parameters were set to standard. PYMOL-v.1.3, a molecular graphic system, was used to visualize discontinuity in the final vaccine construct [52].

### 2.9. Disulfide Engineering

The proper stabilization of the protein construct was verified by covalent disulfide bonds emulating the molecular interaction’s stability by confirming precision geometric conformations. Disulfide bonds were predicted using the disulfide engineering approach on the structure of the target protein. Disulfide engineering was performed for a refined vaccine model with design 2.0. A refined protein model was uploaded for the screening of residue pairs and these pairs were further used in disulfide engineering. The mutated server function was used for mutation with cysteines residues; for this purpose, three pairs were selected [53].

### 2.10. Docking of TLR4 Receptor with Constructed Vaccine Disulfide

After the interaction of the host’s immune cells with the vaccine, an effective immune response is generated. Molecular docking was performed to observe the binding ability of the vaccine with the receptors of the human immune cells. The stimulating and antibacterial immune response was analyzed by observing the TLR4, MHC-I and MHC-II receptors. Later, by employing the HADDOCK-v-2.4 server, the unique protein-to-protein interaction was determined [54]. To model the biomolecular complexes, HADDOCK was used. For the visualization of the docked complexes, PyMOL-v.1.3 was utilized [54]. PDBsum, an online server, was used for analyzing the interactions among docked complexes [55].

### 2.11. Molecular Dynamic Simulation

In each in silico research study, molecular dynamics have an essential role in analyzing the integrity of protein/protein complexes. The essential protein dynamics could be compared to standard nodes to determine a protein’s stability [56,57]. In internal coordinates, collective protein motion was explained through the iMOD (Internal coordinates normal mode analysis) server [58]. The internal motions of the complexes were measured through this server, describing the deformability, covariance, eigenvalues and B-factors. The stiffness of motion indicated the value of standard mode. If the eigenvalue is low, then it helps to deform structures with a specific energy.

### 2.12. Immunogenicity Evaluation of Construct

To validate the immune responses of the modeled vaccine, an in silico immune simulation was carried out by utilizing the online server C-ImmSim-10.1. In the C-ImmSim11 server, three basic components of the functioning mammalian system were replicated, namely, thymus, bone marrow and lymph node [59]. The immune simulation’s input parameters included random-seed (12345), Volume (10) and No of injection-set (1), as well as HLA (A-0101, A-0101, B-0702, B-0702, D-RB1_0101 and DRB1_0101). The existing parameters were fixed as the standard.

### 2.13. Codon Adaptation and In Silico Cloning

It is very important to consider codon optimization because an unadapted codon likely results in an insignificant expression rate in the host. As a result, to maximize gene expression, it should be designed in accordance with the host’s translational machinery. The JCAT (Java Codon Adaptation Tool) was utilized to accommodate the codon vaccination to Ecoli strain K12, a typical prokaryotic model [60]. Furthermore, the GC content, along with CAI, was examined. At the N- and C-termini of the optimized nucleotide sequence, the NcoI and XhoI restriction sites were introduced. Lastly, to validate the production of the targeted vaccine, the optimized DNA sequence of our vaccine construct was cloned (Multiple *E. coli* plasmid pET30a (+) cloning site (MCS)) with Snap Gene 4.3.

## 3. Results

### 3.1. Protein’s Selection

There are 4909 proteins in the whole proteome of K. aerogenes (strain UP000008881). From these, 398 essential proteins were predicted through the online server Geptop-0.5. Total 193 non-homologous proteins were found by removing human homologs by an online analysis via BLASTp. The antigenicity values were used to evaluate these. The best three proteins with the highest antigenicity were preferred for further screening and all of those were extracellular proteins (Table 1).

### 3.2. CTL Epitope Selection and Evaluation

From the target protein of *K. aerogenes,* 39 CTL epitopes (12-mer) were generated (Table S1). The top six epitopes which had high immunogenicity and antigenicity, as well as non-allergenic and non-toxic characteristics, were chosen for the construction of the vaccine, by checking their antigenicity, immunogenicity, allergenicity and toxicity with different tools (Table 2). There was a total of 27 unique HTL epitopes (Table S2). The top three epitopes were chosen for the vaccine by calculating their capability through cytokines (IL-10, IL-4 and IFN-Gamma) (Table 3). Similarly, 15 LBL epitopes were selected (Table S3) from which a total of 5 epitopes was finalized for the synthesis of the vaccine by evaluating their allergenicity, toxicity and immunogenicity (Table 4).

### 3.3. Construction of Vaccine

**Figure 2:**
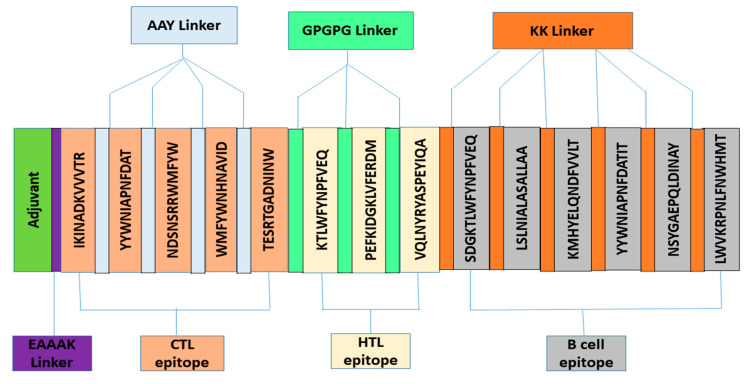
Constructed vaccine figure with adjuvants and linkers.

The vaccine was constructed using all the selected epitopes. The AAY, KK and GPGPG linkers were utilized to attach all MHC-I, LBL and MHC-II epitopes. These linkers were chosen because they help to enhance immunization and epitope presentation, while also preventing the formation of junctional epitopes. Along with the EAAAK linker, cholera enterotoxin subunit B (124 residues) was attached as an adjuvant with the first CTL epitopes. It is noteworthy that EAAAK linkers improve structural stability and reduce the association with neighboring protein regions with effectual division [43]. The final 356-amino acid vaccine built shows how various epitopes and linkers were arranged (Figure 2).

### 3.4. Physiochemical and Immunogenic Profiling

The immunogenic and physiochemical properties of the synthesized vaccine were further studied. When the homology of the produced vaccine was compared to the human proteome, the findings show that no one was similar to any human proteome. After that, we measured our vaccine’s allergenicity, antigenicity and toxicity, which showed our vaccine was extremely antigenic, non-allergenic and non-toxic. ProtParam was utilized to test the physiochemical properties. The pI and MW of the final construct were 9.16 kDa and 40,736.58 kDa, respectively. Our vaccine gave results as follows: the construct had a mean half-life of 30 h in vitro, more than twenty hours in vivo (Yeast) and greater than 10 h in vivo (E. coli); the GRAVY was estimated to be −0.331. All of these characteristics pointed that *K. aerogenes* might act as a promising vaccination candidate.

### 3.5. Structural Evaluation

The vaccine’s secondary structure was analyzed using the PSIPRED and SOPMA servers. The alpha-helix contained 123 residues, contributing to 34.55% of the sequence, 102 residues in extended chains, which accounted for 28.65% of the sequence, and 103 amino acids in coils, which accounted for 28.93% of the construct of the vaccine.

### 3.6. Prediction of Tertiary Structure, Validation and Refinement

**Figure 3:**
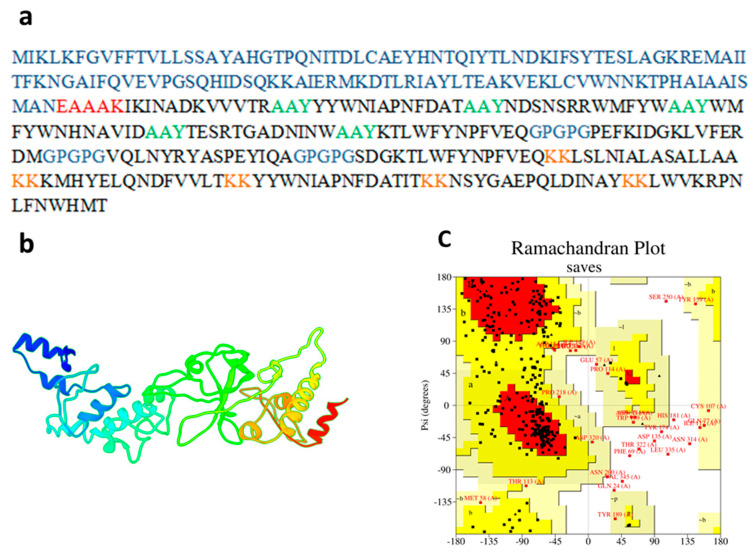
(**a**) Multi-epitope subunit vaccine construct’s sequence, where blue color indicates adjuvant, red color represents EAAAK linker, lime color shows AAY linker, blue color depicts GPGPG, orange color exhibits KK linker and the black color indicates of CTL, HTL, and B-Cell Epitopes (**b**) Refine 3D structure of vaccine construct. (**c**) Ramchandran plot to check the quality of vaccine construct.

I-Tasser, an online server, was utilized to synthesize the tertiary structure of the *K. aerogenes*; the C-score was calculated as −1.66, with this server. Moreover, the vaccine structure was refined by employing the Galaxy-Refine server. According to the enhanced model study, the improved analysis of the model through the Ramachandran plot showed that 60.5% of amino acids were in favored regions, 31.3% in the allowed regions and 3.1% in the outlier regions. The Z-score was calculated as −1.01. The optimized model scored 64.8968 in the ERRAT’s quality-check analysis. These results indicate that the refined model was of excellent quality (see Figure 3a–c).

### 3.7. Selection of B-Cell Epitopes

Antibodies are produced by B-lymphocytes and build humoral immunity [61]. As a result, the vaccine should contain ideal domains of B-cell epitopes. With default parameters, ABCPred 2.0 was used to predict the 3 conformational-discontinuous and 24 linear-continuous epitopes of the vaccine construct. In the vaccine construct, PyMOL v.1.3, a molecular graphic system, was used to visualize the conformational B-cell epitopes.

### 3.8. Disulfide Engineering

**Figure 4:**
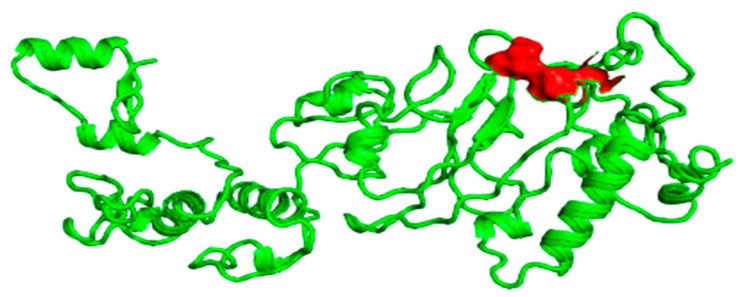
Disulfide engineering indicating the pair energy shown in the sphere form in the red color.

Disulfide engineering was used to improve the stability of the refined vaccine built using Disulfide by Design v-2.0. A pair of 33 residues were used for disulfide engineering. Three residue pairs had energy and Chi3 values within the usual range, so these were chosen for disulfide engineering. As a result, two mutations in the residue pair were formed, including PRO222 and PRO251, with −80.06 and 1.67 kcal/mol of energy, respectively (Figure 4).

### 3.9. Molecular Docking

**Figure 5:**
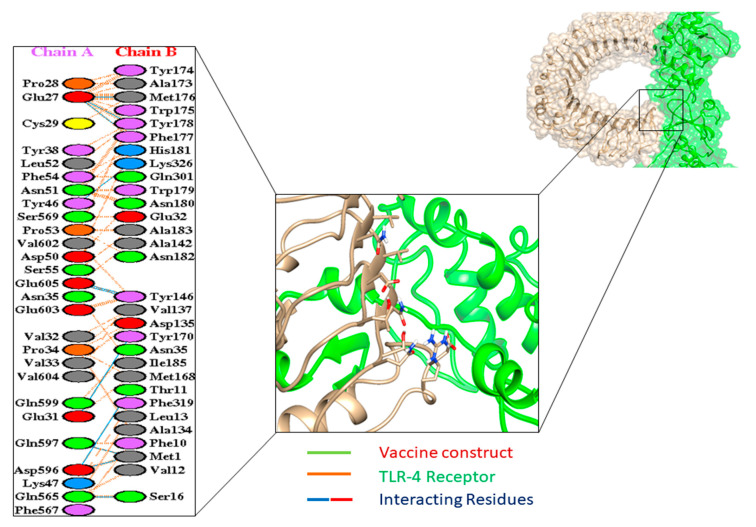
Docked complex of TLR4 receptor with the vaccine receptor indicating the interacting part along with the interacting residue.

For active and immune response, an efficient interaction between the molecules of antigen and the immune receptor is essential. HADDOCK v. 2.4 was used for the docking between vaccine and TLR4, MHC-1 and MHC-II (human immune receptor). In response to bacterial recognition, TLR4 produced an effective immune response. Based on the docking results, the vaccine and TLR4 had a strong interaction. The binding score between TLR4 and the vaccine was calculated as 55.2 kcal/mol. TLR4 had a cyan color, while MEBSV was shown as green on the map (Figure 5). It was determined that there were 10 hydrogen bond interactions between TLR4 and the vaccine, within a range of 3.34 Å.

**Figure 6:**
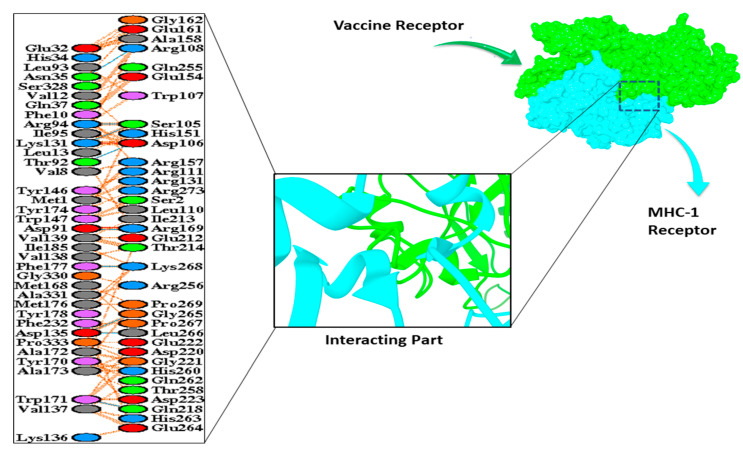
Interaction of vaccine receptor with MHC-I receptor.

**Figure 7:**
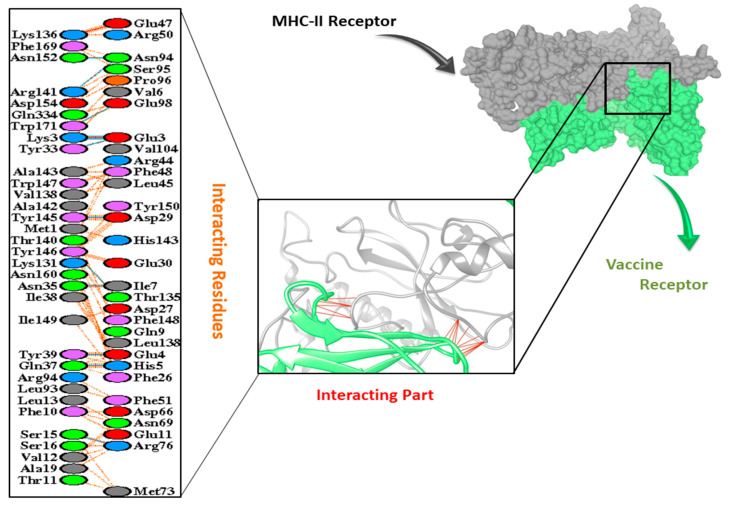
Interaction of vaccine receptor with MHC-II receptor.

Moreover, the vaccine structure was also docked with the MHC1 and MHC II receptors using HADDOCK V.2.4. The results showed a HADDOCK score of 169.1 and 131.8 with the desolation energy of −36.3 (kcal/mol) and −53.6 (kcal/mol), respectively. The docking results are shown in Table 5. A total of 11 Hydrogen bond interactions were observed in the docked complex of MHC-II with the vaccine construct, although the interacting residues observed in the MHC-I complex were 7, as shown in Figure 6 and Figure 7.

### 3.10. Molecular Dynamic Simulation

**Figure 8:**
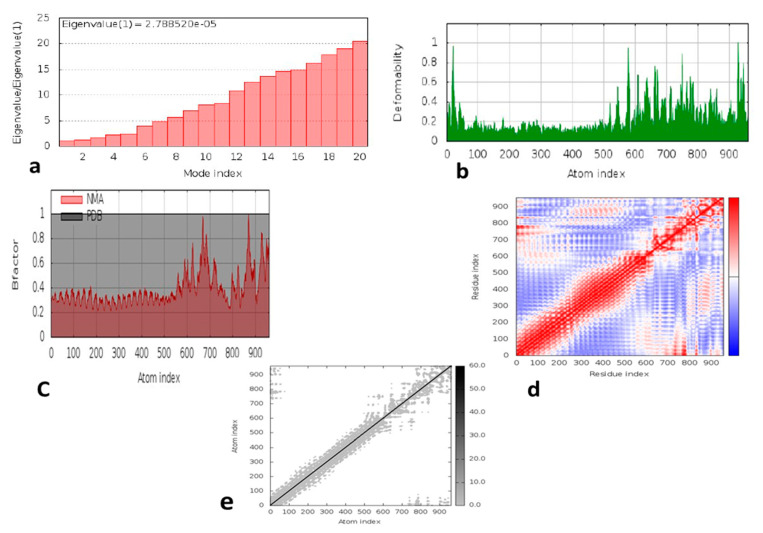
Molecular dynamic simulation of the vaccine–TLR4 complex, showing: (**a**) eigenvalue; (**b**) deformability; (**c**) B-factor; (**d**) covariance matrix; (**e**) elastic network analysis.

Protein mobility and stabilization were examined using a large-scale normal mode analysis (NMA). Relying on internal coordinates of the docked complex, the iMODS-server was used for this evaluation. The deformability of the complex was determined by the distortion of the individual residue, as is shown by the hinges of the chain (Figure 8b). The value of the complex was calculated as 2.788520 × 10^5^, (Figure 8a). The eigenvalue of each normal mode was determined by reversing the variance associated with it [62]. The value of the B-factor was proportional to RMS due to the normal mode analysis (Figure 8c). A covariance matrix represents the pairings of the residue pairs, with different pairs of correlated, dis-associated, or irrelevant motions represented in different colors such as reddish, bluish and white (Figure 8d). The elastic map depicts joint atoms linked by springs, with every single point showing one spring and the grey color representing stiffer areas, with intensity being proportional to stiffness (Figure 8e).

### 3.11. Immune-Simulation

**Figure 9:**
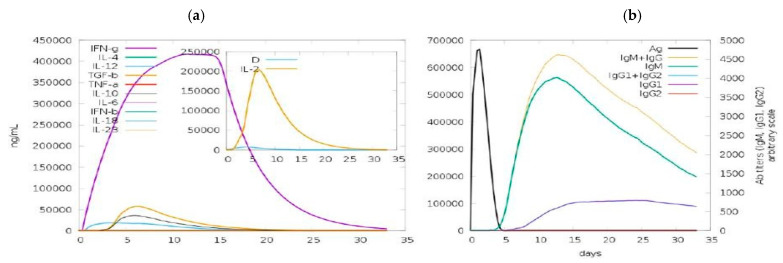
In silico immune responses of the vaccine as an antigen: (**a**) immunoglobulin generation and B-cell isotypes following exposure in different states with the Simpson index to the antigen; (**b**) development of cytokine, interleukins.

For specific immune responses against a pathogen, every secondary and primary immune response played an important role. The in silico host immune system responses to the antigen are shown in Figure 7. The primary reaction was described by the high concentration of IgG + IgG and IgM, followed, in the secondary and primary phases, by IgM, IgG1 + IgG2 and IgG1 with consequent antigen reduction. The interleukin and cytokine reactions were found to be extremely effective. A successful immune response to the vaccine was observed, as well as clearance after subsequent experiences, as shown in Figure 9.

### 3.12. In Silico Cloning

**Figure 10:**
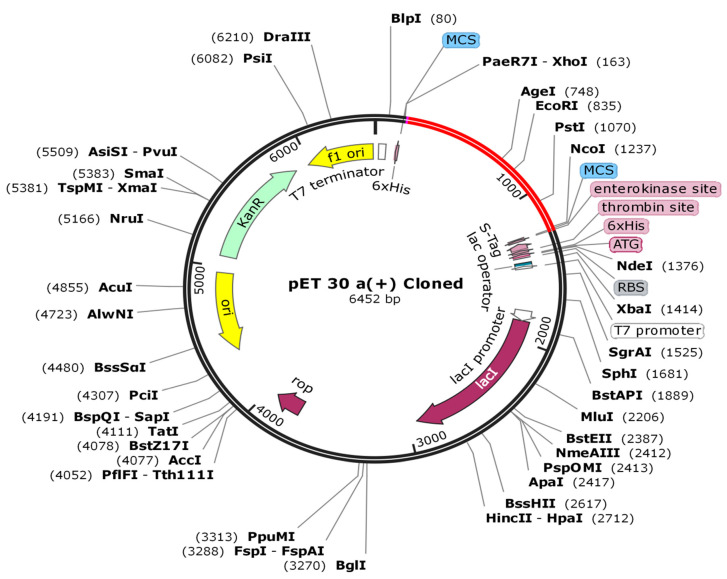
In silico cloning of codon-optimized vaccine into *E. coli* K12’s expression system. The plasmid backbone is shown in black, while the inserted DNA sequence is shown in red.

To ensure that the vaccine protein was expressed efficiently in the *E. coli* host system, codon optimization and in silico cloning were performed. The codons used in the vaccine structure were matched to the prospective host *E. coli* K12’s codon. The CAI score was 0.9 and the GC content was 48.87% in the improved DNA. A CAI with a value of 1.o was proposed as a good compromise. The synthesized codon was inserted among Nco1 and the restriction sites in the *E. coli* vector pET 30a (+) (Figure 10). Furthermore, the size of the clone was 6452 bp.

## 4. Discussion

Even though immunological experiments are the most powerful immune modalities, developing a vaccine based on them is pretty time-consuming and costly [63,64]. However, recent developments in immunological bioinformatics have led to valuable tools and resources that can speed up and lower the cost of traditional vaccine production [65]. Immuno-informatic methods for predicting appropriate antigenic epitopes of the specific antibody are essential to developing a multi-epitope subunit vaccine [66,67]. Effective T- and B-cell epitopes extracted from *Klebsiella aerogenes*’s highest antigenic *Enterobacteriales* family protein were utilized to create a multi-epitope vaccine in the current research study.

The mortality rate remains from 20 to 50 percent in patients with Gram-negative *bacteremia* [68]. *K. aerogenes* was the second most common cause of Gram-negative *bacteremia* in patients infected in England and Wales, behind only *Escherichia coli* [69]. There is currently no specific immunological remedy for *Klebsiella* pathogens, though immunological therapies for other Gram-negative bacilli obtained in hospitals have been successfully developed [70,71].

The proposed epitopes are used in an optimal multi-epitope vaccine to stimulate a full network of immune responses [72]. Keeping this in view, both forms of epitopes were predicted from the *Klebsiella* protein and analyzed in detail. For broad-spectrum functionality, the number of HLA binding alleles was also assessed. Overlapping epitopes, on the other hand, were omitted from the final collection to maintain epitopic heterogeneity in the vaccine protein. The epitopes that were chosen were then used to create a vaccine. A vaccine’s efficacy is determined by the population in which it is administered [73]. The findings revealed that the considered epitopes and alleles are present in important parts of the globe, with a population coverage of 89.29 percent. The vaccine consists of 356 amino acid residues.

Furthermore, the linkers were attached, which helped in the functional stabilization within all epitopes for their transfer in organisms, allowing them to function independently. The epitope vaccine was considered highly antigenic, immunogenic and non-allergenic, demonstrating its ability to stimulate the immune system without provoking allergies. Moreover, the vaccine construct showed a molecular weight of 40,736.58 kDa and showed no similarity with the human proteome. Besides this, in the E.coli host’s overexpression, the recombinant vaccine protein demonstrated excellent solubility, making it simple to enter the host [74].

Three-dimensional structure modeling provides a wealth of knowledge about the spatial arrangement of important protein components and assists in analyzing ligand interaction, protein function, dynamics and other proteins. The vaccine construct’s beneficial properties significantly improved after refinement. To find errors in the modeled vaccine design, different structure validation methods were applied. Cells, phagocytes and the development of inflammatory cytokines, i.e., tumor necrosis factor-alpha (TNF-), which increase host cells’ apoptosis and necrosis, were all triggered when *Klebsiella aerogenes* binds with the TLR4 receptor [75,76]. The molecular docking and molecular dynamic simulation study revealed a strong interaction between the vaccine and TLR-4, MHC-1 and MHC-II, as well as the fact that this balanced bonding took very little energy. These findings strongly suggest that the vaccine can successfully bind to immune receptors. Hypothetically, the vaccine can elicit humoral as well as cellular immune responses. Our vaccine produced IFN-γ with the highest levels of IL-10 and IL-2 activity. Extracellular defense against *Klebsiella aerogenes* was provided by antibodies. Furthermore, the Simpson Index (D) recommends a number of immune responses which can be considered as a subunit vaccine because they include many T- and B-cell epitopes. The serological assessment of immunoreactivity is a vital step in the validation of a vaccine’s structure. Since mRNA codons are incompatible, the translational output of foreign genes can vary within the target host, which requires codon optimization for increased expression [77]. The reported CAI amount of 0.9 was nearly 1.0 and the GC was 48.87% present, which is within the ideal range (30–70%), suggesting that a higher output within the *E. coli* K12’s system was possible [78]. In silico cloning was conducted using the pET30a (+) vector as a baseline for future vaccine synthesis. This plasmid contains both His-tag and S-tag as fusion partners, making purification easier.

Furthermore, the concentration of charged and polar residues in the S-tag sequence enhances protein stability [79]. The reverse vaccinology technique used in this study will aid in the development of *K. aerogenes* strain-specific treatments. Despite considerable success in the development of medicines to combat *K. aerogenes* infections until now, a safer and efficient vaccination to limit the spread of this opportunistic pathogen is highly needed. The pET30a (+) vector was used for in silico cloning as a recommendation for future vaccine manufacturing. A plasmid with both His-tag and S-tag as fusion partners makes purification easier.

According to our current study, the multiepitope-based subunit vaccine has outstanding characteristics that give it an edge over previous vaccines. It contains B-cell, CTL and HTL epitopes; thus, it may induce humoral and cellular resistance in the host. It is composed of epitopes that target various HLAs and enable it to determine new T-cell receptors that have been shown to be effective across a large population. Several immunogenic regions seem to fuse into one peptide fragment so that a single vaccine could target several proteins. This increases the vaccine’s efficacy. As a result, it is an excellent candidate to develop a vaccine against *Klebsiella aerogenes* infections.

## 5. Conclusions

The infection caused by *K. aerogenes* has long been a mystery, but it is now a global health concern. It is still impossible to treat it or prevent it with medication or vaccines. Antibacterial medications have been explored, but none have proven to be effective in preventing infection. The main goal of building an MEBSV is to use subtractive genomics and immuno-informatic approaches to regulate humoral and cellular immune responses. The therapeutic proteins required for bacterial viability but lacking in the host were identified using a subtractive genomics technique. The proposed MEBSV model, when combined with computer analyses and immune-information data, could aid in the development of a possible vaccine against *K. aerogenes* infection. Our research study, on the other hand, is the result of a complete immunization strategy. As a result, additional laboratory studies are required to demonstrate the MEBSV model’s efficacy and safety.

## Figures and Tables

**Table 1:** Top selected proteins with highest antigenic nature with extra-cellular location.

Sr. No	Protein Name	Accession No	Antigenicity	Helices	Location
1	Lipopolysaccharide export system protein LptA	A0A0H3FMX7	0.77	0	Extracellular
2	LPS-assembly protein LptD	A0A0H3FNQ6	0.67	0	Extracellular
3	Outer-membrane lipoprotein carrier protein	A0A0H3FTL0	0.71	0	Extracellular

**Table 2:** Final CTL selected epitopes for the construction of the vaccine against *Klebsiella aerogenes*.

Epitope	Protein	Alleles	Position	Antigenicity	Immunogenicity
IKINADKVVVTR	Lipopolysaccharide export system protein LptA	HLA-A*31:01	65–76	0.9404	0.02297
YYWNIAPNFDAT	LPS-assembly protein LptD	HLA-A*23:01 HLA-C*07:02 HLA-C*14:02 HLA-A*24:02	247–258	1.9021	0.36777
NDSNSRRWMFYW	LPS-assembly protein LptD	HLA-B*57:01 HLA-B*58:01 HLA-A*01:01	304–315	1.8656	0.02653
WMFYWNHNAVID	LPS-assembly protein LptD	HLA-C*07:02 HLA-B*39:01 HLA-B*48:01	311–322	1.3380	0.41831
TESRTGADNINW	LPS-assembly protein LptD	HLA-B*44:02 HLA-B*44:03 HLA-B*57:01	583–594	1.3921	0.23133
KTLWFYNPFVEQ	Outer-membrane lipoprotein carrier protein	HLA-A*02:01 HLA-A*32:01	85–96	0.5371	0.51613

**Table 3:** Final HTl epitopes for the construction of the vaccine against *Klebsiella aerogenes*.

Epitope	Protein	Alleles	Position	Antigenicity	IFN-Y	IL-4	IL-10
PEFKIDGKLVFERDM	LPS-assembly protein LptD	HLA-DRB1*03:09 HLA-DRB1*03:05 HLA-DRB1*03:01 HLA-DRB1*03:06 HLA-DRB1*03:07 HLA-DRB1*03:08 HLA-DRB1*11:28 HLA-DRB1*13:05 HLA-DRB1*11:07	483–497	0.9915	Positive	Inducer	Negative
VQLNYRYASPEYIQA	LPS-assembly protein LptD	HLA-DRB1*09:01 HLA-DRB1*07:03 HLA-DRB1*11:02 HLA-DRB1*11:21 HLA-DRB1*13:22	649–663	1.1174	Positive	Inducer	Negative
SDGKTLWFYNPFVEQ	Outer-membrane lipoprotein carrier protein	HLA-DQA1*01:01/DQB1*05:01	82–96	0.6977	Positive	Inducer	Negative

**Table 4:** Final selected B-cell epitopes for vaccine construction.

Epitope	Protein	Score	Position	Antigenicity	Immunogenicity
LSLNIALASALLAA	Lipopolysaccharide export system protein LptA	8	0.58	1.0434	0.06495
KMHYELQNDFVVLT	Lipopolysaccharide export system protein LptA	111	0.52	0.9433	0.1772
YYWNIAPNFDATIT	LPS-assembly protein LptD	247	0.85	1.7077	0.49894
NSYGAEPQLDINAY	LPS-assembly protein LptD	386	0.68	1.7424	0.19838
LWVKRPNLFNWHMT	Outer-membrane lipoprotein carrier protein	60	0.68	1.1223	0.05981

**Table 5:** Docking table indicating the docking scores with different energy values of TlR-4, MHC-I and MHC-II with the vaccine construct.

Parameters	Values
**TLR-4**	
HADDOCK-v.2.2 score	88.6 ± 20.9
Cluster size	8
RMSD from the overall lowest energy structure	6.4 ± 0.3
Van-der-Waals energy	−107.4 ± 11.7
Electrostatic energy	−129.9 ± 26.6
Desolvation energy	−68.9 ± 5.7
Restraint violation energy	2909.2 ± 318.20
Buried surface area	3238.0 ± 127.9
Z-score	−0.1
**MHC-1 Receptor**	
HADDOCK-v.2.4 score	169.1 ± 20.3
Cluster size	7
RMSD from the overall lowest energy structure	18.7 ± 0.0
Van-der-Waals energy	−125.6 ± 10.0
Electrostatic energy	−246.3 ± 39.8
Desolvation energy	−36.3 ± 4.9
Restraint violation energy	3803.0 ± 191.5
Buried surface area	4018.7 ± 264.0
Z-score	−1.2
**MHC-II Receptor**	
HADDOCK-v.2.4 score	131.8 ± 26.7
Cluster size	4
RMSD from the overall lowest energy structure	16.3 ± 0.2
Van-der-Waals energy	−94.5 ± 15.4
Electrostatic energy	−301.3 ± 34.0
Desolvation energy	−53.6 ± 4.1
Restraint violation energy	3401.9 ± 175.4
Buried surface area	3492.0 ± 266.7
Z-score	−1.7

## Data Availability

The data presented in this study are available within the article.

## Supplementary Materials

The following are available online at https://www.mdpi.com/article/10.3390/vaccines9111373/s1, Table S1: Final MHC-I epitopes selected on the basis of different parameters, Table S2: Final MHC-II epitopes selected on the basis of different parameters, Table S3: Final B Cell epitopes selected on the basis of different parameters.

## References

[1] Tindall B., Sutton G., Garrity G. (2017). Enterobacter aerogenes Hormaeche and Edwards 1960 (Approved Lists 1980) and Klebsiella mobilis Bascomb et al. 1971 (Approved Lists 1980) share the same nomenclatural type (ATCC 13048) on the Approved Lists and are homotypic synonyms, with consequences for the name Klebsiella mobilis Bascomb et al. 1971 (Approved Lists 1980). Int. J. Syst. Evol. Microbiol..

[2] Diene S.M., Merhej V., Henry M., El Filali A., Roux V., Robert C., Azza S., Gavory F., Barbe V., La Scola B. (2013). The rhizome of the multidrug-resistant Enterobacter aerogenes genome reveals how new “killer bugs” are created because of a sympatric lifestyle. Mol. Biol. Evol..

[3] Davin-Regli A. (2015). Enterobacter aerogenes and Enterobacter cloacae; versatile bacterial pathogens confronting antibiotic treatment. Front. Microbiol..

[4] Guh A.Y., Bulens S.N., Mu Y., Jacob J.T., Reno J., Scott J., Wilson L.E., Vaeth E., Lynfield R., Shaw K.M. (2015). Epidemiology of carbapenem-resistant Enterobacteriaceae in 7 US communities, 2012–2013. JAMA.

[5] Lee H.-J., Choi J.-K., Cho S.-Y., Kim S.-H., Park S.-H., Choi S.-M., Lee D.-G., Choi J.-H., Yoo J.-H. (2016). Carbapenem-resistant Enterobacteriaceae: Prevalence and risk factors in a single community-based hospital in Korea. Infect. Chemother..

[6] Robert J., Pantel A., Mérens A., Lavigne J., Nicolas-Chanoine M. (2014). On behalf of ONERBA’s carbapenem resistance study group. Incidence rates of carbapenemase-producing Enterobacteriaceae clinical isolates in France: A prospective nationwide study in 2011–12. J. Antimicrob. Chemother..

[7] Franolić I., Bedenić B., Beader N., Lukić-Grlić A., Mihaljević S., Bielen L., Zarfel G., Meštrović T. (2019). NDM-1-producing Enterobacter aerogenes isolated from a patient with a JJ ureteric stent in situ. CEN Case Rep..

[8] Khajuria A., Praharaj A.K., Kumar M., Grover N. (2014). Carbapenem resistance among Enterobacter species in a tertiary care hospital in central India. Chemother. Res. Pract..

[9] Wyres K.L., Holt K.E. (2016). Klebsiella pneumoniae population genomics and antimicrobial-resistant clones. Trends Microbiol..

[10] Gomez-Simmonds A., Annavajhala M.K., Wang Z., Macesic N., Hu Y., Giddins M.J., O’Malley A., Toussaint N.C., Whittier S., Torres V.J. (2018). Genomic and geographic context for the evolution of high-risk carbapenem-resistant Enterobacter cloacae complex clones ST171 and ST78. MBio.

[11] Jabri E., Carr M.B., Hausinger R.P., Karplus P.A. (1995). The crystal structure of urease from Klebsiella aerogenes. Science.

[12] Corona F., Blanco P., Alcalde-Rico M., Hernando-Amado S., Lira F., Bernardini A., Sanchez M.B., Martinez J.L. (2016). The analysis of the antibiotic resistome offers new opportunities for therapeutic intervention. Future Med. Chem..

[13] Martinez J.L., Fajardo A., Garmendia L., Hernandez A., Linares J.F., Martínez-Solano L., Sánchez M.B. (2008). A global view of antibiotic resistance. FEMS Microbiol. Rev..

[14] Passarelli-Araujo H., Palmeiro J.K., MOHARANA K.C., Pedrosa-Silva F., Dalla-Costa L.M., Venancio T.M. (2019). Molecular epidemiology of 16S rRNA methyltransferase in Brazil: RmtG in Klebsiella aerogenes ST93 (CC4). Anais Acad. Bras. Ciênc..

[15] Grazziotin A.L., Vidal N.M., Palmeiro J.K., Dalla-Costa L.M., Venancio T.M. (2016). Genome sequencing of four multidrug-resistant Enterobacter aerogenes isolates from hospitalized patients in Brazil. Front. Microbiol..

[16] Ma D.-Y., Huang H.-Y., Zou H., Wu M.-L., Lin Q.-X., Liu B., Huang S.-F. (2020). Carbapenem-Resistant Klebsiella aerogenes Clinical Isolates from a Teaching Hospital in Southwestern China: Detailed Molecular Epidemiology, Resistance Determinants, Risk Factors and Clinical Outcomes. Infect. Drug Resist..

[17] Ahmad B., Ashfaq U.A., Rahman M.U., Masoud M.S., Yousaf M.Z. (2019). Conserved B and T cell epitopes prediction of ebola virus glycoprotein for vaccine development: An immuno-informatics approach. Microb. Pathog..

[18] Aldakheel F.M., Abrar A., Munir S., Aslam S., Allemailem K.S., Khurshid M., Ashfaq U.A. (2021). Proteome-Wide Mapping and Reverse Vaccinology Approaches to Design a Multi-Epitope Vaccine against Clostridium perfringens. Vaccines.

[19] Ashfaq U.A., Saleem S., Masoud M.S., Ahmad M., Nahid N., Bhatti R., Almatroudi A., Khurshid M. (2021). Rational design of multi epitope-based subunit vaccine by exploring MERS-COV proteome: Reverse vaccinology and molecular docking approach. PLoS ONE.

[20] Aslam S., Ahmad S., Noor F., Ashfaq U.A., Shahid F., Rehman A., Tahir Ul Qamar M., Alatawi E.A., Alshabrmi F.M., Allemailem K.S. (2021). Designing a Multi-Epitope Vaccine against Chlamydia trachomatis by Employing Integrated Core Proteomics, Immuno-Informatics and In Silico Approaches. Biology.

[21] Khalid H., Ashfaq U.A. (2020). Exploring HCV genome to construct multi-epitope based subunit vaccine to battle HCV infection: Immunoinformatics based approach. J. Biomed. Inform..

[22] Mahmood M., Javaid A., Shahid F., Ashfaq U.A. (2021). Rational design of multimeric based subunit vaccine against Mycoplasma pneumonia: Subtractive proteomics with immunoinformatics framework. Infect. Genet. Evol.

[23] Rehman A., Ahmad S., Shahid F., Albutti A., Alwashmi A.S.S., Aljasir M.A., Alhumeed N., Qasim M., Ashfaq U.A., Tahir Ul Qamar M. (2021). Integrated Core Proteomics, Subtractive Proteomics, and Immunoinformatics Investigation to Unveil a Potential Multi-Epitope Vaccine against Schistosomiasis. Vaccines.

[24] Shahid F., Ashfaq U.A., Javaid A., Khalid H. (2020). Immunoinformatics guided rational design of a next generation multi epitope based peptide (MEBP) vaccine by exploring Zika virus proteome. Infect. Genet. Evol.

[25] Kalita P., Lyngdoh D.L., Padhi A.K., Shukla H., Tripathi T. (2019). Development of multi-epitope driven subunit vaccine against Fasciola gigantica using immunoinformatics approach. Int. J. Biol. Macromol..

[26] Azhagesan K., Ravindran B., Raman K. (2018). Network-based features enable prediction of essential genes across diverse organisms. PLoS ONE.

[27] Doytchinova I.A., Flower D.R. (2007). VaxiJen: A server for prediction of protective antigens, tumour antigens and subunit vaccines. BMC Bioinform..

[28] Krogh A., Larsson B., Von Heijne G., Sonnhammer E.L. (2001). Predicting transmembrane protein topology with a hidden Markov model: Application to complete genomes. J. Mol. Biol..

[29] Moutaftsi M., Peters B., Pasquetto V., Tscharke D.C., Sidney J., Bui H.-H., Grey H., Sette A. (2006). A consensus epitope prediction approach identifies the breadth of murine T CD8+-cell responses to vaccinia virus. Nat. Biotechnol..

[30] Gupta S., Kapoor P., Chaudhary K., Gautam A., Kumar R., Raghava G.P., Consortium O.S.D.D. (2013). In silico approach for predicting toxicity of peptides and proteins. PLoS ONE.

[31] Nielsen M., Lundegaard C., Lund O. (2007). Prediction of MHC class II binding affinity using SMM-align, a novel stabilization matrix alignment method. BMC Bioinform..

[32] Zhu J., Paul W.E. (2008). CD4 T cells: Fates, functions, and faults. Blood J. Am. Soc. Hematol..

[33] Wang P., Sidney J., Kim Y., Sette A., Lund O., Nielsen M., Peters B. (2010). Peptide binding predictions for HLA DR, DP and DQ molecules. BMC Bioinform..

[34] Dhanda S.K., Vir P., Raghava G.P. (2013). Designing of interferon-gamma inducing MHC class-II binders. Biol. Direct.

[35] Dhanda S.K., Gupta S., Vir P., Raghava G. (2013). Prediction of IL4 inducing peptides. Clin. Dev. Immunol..

[36] Nagpal G., Usmani S.S., Dhanda S.K., Kaur H., Singh S., Sharma M., Raghava G.P. (2017). Computer-aided designing of immunosuppressive peptides based on IL-10 inducing potential. Sci. Rep..

[37] Cooper M.D. (2015). The early history of B cells. Nat. Rev. Immunol..

[38] Saha S., Raghava G.P.S. (2006). Prediction of continuous B-cell epitopes in an antigen using recurrent neural network. Proteins Struct. Funct. Bioinform..

[39] Dar H.A., Zaheer T., Shehroz M., Ullah N., Naz K., Muhammad S.A., Zhang T., Ali A. (2019). Immunoinformatics-aided design and evaluation of a potential multi-epitope vaccine against Klebsiella pneumoniae. Vaccines.

[40] Bui H.-H., Sidney J., Dinh K., Southwood S., Newman M.J., Sette A. (2006). Predicting population coverage of T-cell epitope-based diagnostics and vaccines. BMC Bioinform..

[41] Nain Z., Abdulla F., Rahman M.M., Karim M.M., Khan M.S.A., Sayed S.B., Mahmud S., Rahman S.R., Sheam M.M., Haque Z. (2020). Proteome-wide screening for designing a multi-epitope vaccine against emerging pathogen Elizabethkingia anophelis using immunoinformatic approaches. J. Biomol. Struct. Dyn..

[42] Li W., Joshi M.D., Singhania S., Ramsey K.H., Murthy A.K. (2014). Peptide vaccine: Progress and challenges. Vaccines.

[43] Arai R., Ueda H., Kitayama A., Kamiya N., Nagamune T. (2001). Design of the linkers which effectively separate domains of a bifunctional fusion protein. Protein Eng..

[44] Nezafat N., Ghasemi Y., Javadi G., Khoshnoud M.J., Omidinia E. (2014). A novel multi-epitope peptide vaccine against cancer: An in silico approach. J. Theor. Biol..

[45] Mahram A., Herbordt M.C. Fast and accurate NCBI BLASTP: Acceleration with multiphase FPGA-based prefiltering. Proceedings of the 24th ACM International Conference on Supercomputing.

[46] Walker J.M. (2005). The Proteomics Protocols Handbook.

[47] Zheng W., Li Y., Zhang C., Pearce R., Mortuza S., Zhang Y. (2019). Deep-learning contact-map guided protein structure prediction in CASP13. Proteins Struct. Funct. Bioinform..

[48] Heo L., Park H., Seok C. (2013). GalaxyRefine: Protein structure refinement driven by side-chain repacking. Nucleic Acids Res..

[49] Lovell S.C., Davis I.W., Arendall III W.B., De Bakker P.I., Word J.M., Prisant M.G., Richardson J.S., Richardson D.C. (2003). Structure validation by Cα geometry: ϕ, ψ and Cβ deviation. Proteins Struct. Funct. Bioinform..

[50] Lengths M., Angles M. (2018). Limitations of structure evaluation tools errat. Quick Guidel. Comput. Drug Des..

[51] Ponomarenko J., Bui H.-H., Li W., Fusseder N., Bourne P.E., Sette A., Peters B. (2008). ElliPro: A new structure-based tool for the prediction of antibody epitopes. BMC Bioinform..

[52] Craig D.B., Dombkowski A.A. (2013). Disulfide by Design 2.0: A web-based tool for disulfide engineering in proteins. BMC Bioinform..

[53] Van Zundert G., Rodrigues J., Trellet M., Schmitz C., Kastritis P., Karaca E., Melquiond A., van Dijk M., De Vries S., Bonvin A. (2016). The HADDOCK2. 2 web server: User-friendly integrative modeling of biomolecular complexes. J. Mol. Biol..

[54] Laskowski R.A. (2009). PDBsum new things. Nucleic Acids Res..

[55] Van Aalten D.M., De Groot B.L., Findlay J.B., Berendsen H.J., Amadei A. (1997). A comparison of techniques for calculating protein essential dynamics. J. Comput. Chem..

[56] Wüthrich K., Wagner G., Richarz R., Braun W. (1980). Correlations between internal mobility and stability of globular proteins. Biophys. J..

[57] López-Blanco J.R., Aliaga J.I., Quintana-Ortí E.S., Chacón P. (2014). iMODS: Internal coordinates normal mode analysis server. Nucleic Acids Res..

[58] Rapin N., Lund O., Bernaschi M., Castiglione F. (2010). Computational immunology meets bioinformatics: The use of prediction tools for molecular binding in the simulation of the immune system. PloS ONE.

[59] Grote A., Hiller K., Scheer M., Münch R., Nörtemann B., Hempel D.C., Jahn D. (2005). JCat: A novel tool to adapt codon usage of a target gene to its potential expression host. Nucleic Acids Res..

[60] Lund F.E. (2008). Cytokine-producing B lymphocytes—Key regulators of immunity. Current Opin. Immunol..

[61] Kovacs J.A., Chacón P., Abagyan R. (2004). Predictions of protein flexibility: First-order measures. Proteins Struct. Funct. Bioinform..

[62] Nezafat N., Karimi Z., Eslami M., Mohkam M., Zandian S., Ghasemi Y. (2016). Designing an efficient multi-epitope peptide vaccine against Vibrio cholerae via combined immunoinformatics and protein interaction based approaches. Comput. Biol. Chem..

[63] Kazi A., Chuah C., Majeed A.B.A., Leow C.H., Lim B.H., Leow C.Y. (2018). Current progress of immunoinformatics approach harnessed for cellular-and antibody-dependent vaccine design. Pathog. Glob. Health.

[64] Plotkin S., Robinson J.M., Cunningham G., Iqbal R., Larsen S. (2017). The complexity and cost of vaccine manufacturing—An overview. Vaccine.

[65] Yin D., Li L., Song X., Li H., Wang J., Ju W., Qu X., Song D., Liu Y., Meng X. (2016). A novel multi-epitope recombined protein for diagnosis of human brucellosis. BMC Infect. Dis..

[66] Cherryholmes G.A., Stanton S.E., Disis M.L. (2015). Current methods of epitope identification for cancer vaccine design. Vaccine.

[67] Zinner S., Peter G. (1983). The potential role of cell wall core glycolipids in the immunoprophylaxis and immunotherapy of Gram-negative rod bacteraemia. Med. Microbiol..

[68] Young S.E. (1982). Bacteraemia 1975–1980: A survey of cases reported to the PHLS Communicable Disease Surveillance Centre. J. Infect..

[69] Cryz S. (1983). Progress in immunization against Klebsiella infections. Eur. J. Clin. Microbiol..

[70] Ziegler E.J., McCutchan J.A., Fierer J., Glauser M.P., Sadoff J.C., Douglas H., Braude A.I. (1982). Treatment of gram-negative bacteremia and shock with human antiserum to a mutant Escherichia coli. N. Engl. J. Med..

[71] Lin X., Chen S., Xue X., Lu L., Zhu S., Li W., Chen X., Zhong X., Jiang P., Sename T.S. (2016). Chimerically fused antigen rich of overlapped epitopes from latent membrane protein 2 (LMP2) of Epstein–Barr virus as a potential vaccine and diagnostic agent. Cell. Mol. Immunol..

[72] Phillips R.O., Phanzu D.M., Beissner M., Badziklou K., Luzolo E.K., Sarfo F.S., Halatoko W.A., Amoako Y., Frimpong M., Kabiru A.M. (2015). Effectiveness of routine BCG vaccination on buruli ulcer disease: A case-control study in the Democratic Republic of Congo, Ghana and Togo. PLoS Negl. Trop. Dis..

[73] Khatoon N., Pandey R.K., Prajapati V.K. (2017). Exploring Leishmania secretory proteins to design B and T cell multi-epitope subunit vaccine using immunoinformatics approach. Sci. Rep..

[74] Brennan M.J. (2017). The enigmatic PE/PPE multigene family of mycobacteria and tuberculosis vaccination. Infect. Immun..

[75] Dheenadhayalan V., Delogu G., Brennan M.J. (2006). Expression of the PE_PGRS 33 protein in Mycobacterium smegmatis triggers necrosis in macrophages and enhanced mycobacterial survival. Microbes Infect..

[76] Pandey R.K., Bhatt T.K., Prajapati V.K. (2018). Novel immunoinformatics approaches to design multi-epitope subunit vaccine for malaria by investigating anopheles salivary protein. Sci. Rep..

[77] Shey R.A., Ghogomu S.M., Esoh K.K., Nebangwa N.D., Shintouo C.M., Nongley N.F., Asa B.F., Ngale F.N., Vanhamme L., Souopgui J. (2019). In-silico design of a multi-epitope vaccine candidate against onchocerciasis and related filarial diseases. Sci. Rep..

[78] Hengen P.N. (1995). Purification of His-Tag fusion proteins from Escherichia coli. Trends Biochem. Sci..

[79] Raines R.T., McCormick M., Van Oosbree T.R., Mierendorf R.C. (2000). [23] The S· tag fusion system for protein purification. Methods Enzymol..

