# Supplementary material for: Development of a Candidate Multi-Epitope Subunit Vaccine against Klebsiella aerogenes: Subtractive Proteomics and Immuno-Informatics Approach
Source: Vaccines (Basel). 2021 Nov 22;9(11):1373. doi: 10.3390/vaccines9111373 (PMC8624419; doi:10.3390/vaccines9111373)
Supplement: Supplementary file 1 [file vaccines-09-01373-s001.zip › vaccines-1446041-supplementary.pdf]

Table S1: Final MHC-I epitopes selected on the basis of different parameters

Protein	Epitopes	Position	alleles	antigenicity	immunogenicity
**Lipopolysaccharide export system protein LptA**	IKINADKVVVTR	65-76	HLA-A*31:01	**0.9404**	0.02297
**LPS-assembly protein LptD**	NDSNSRRWMFYW	304-315	HLA-B*57:01 HLA-B*58:01 HLA-A*01:01	**1.8656**	0.02653
	GMTASWPIVDRW	682-693	HLA-B*57:01 HLA-B*58:01	**0.5632**	0.31319
	DQSHIGAYDSTL	518-529	HLA-B*38:01 HLA-B*39:01	**0.5389**	0.07989
	TIEYRRDENRLV	638-649	HLA-C*06:02 HLA-C*07:01	**0.8465**	0.27979
	SNSRRWMFYWNH	306-317	HLA-B*57:01 HLA-B*58:01	**0.8565**	0.38716
	IEYRRDENRLVQ	639-650	HLA-C*06:02 HLA-C*07:01	**0.7103**	0.27756
	EYRRDENRLVQL	640-651	HLA-C*06:02 HLA-C*07:01	**0.6323**	0.1888
	FDATITPHYMNK	255-266	HLA-A*11:01 HLA-A*03:01	**0.7156**	0.0844
	QVGMTASWPIVD	680-691	HLA-E*01:01 HLA-A*32:01	**0.7646**	0.0908
	SQVGMTASWPIV	679-690	HLA-E*01:01 HLA-A*32:01 HLA-B*39:01	**1.1210**	0.06821

	LMKQRGENRYTI	153-164	HLA-B*08:01 HLA-E*01:01	**0.6869**	0.05632
	RRWMFYWNHNAV	309-320	HLA-B*27:05 HLA-A*02:01 HLA-B*39:01	**0.8355**	0.26285
	QVAEIWNARFKL	194-205	HLA-B*40:01 HLA-A*32:01 HLA-A*26:01 HLA-A*25:01 HLA-B*40:02	**0.8663**	0.51207
	NSRRWMFYWNHN	307-318	HLA-B*57:01	**0.7878**	0.44593
	YYWNIAPNFDAT	247-258	HLA-A*23:01 HLA-C*07:02 HLA-C*14:02 HLA-A*24:02	**1.9021**	0.36777
	VERFNISVGQIY	569-590	HLA-B*44:03 HLA-A*30:02 HLA-B*44:02	**0.6663**	0.1489
	NTFGINIELRGL	748-759	HLA-A*68:02 HLA-A*68:01	**2.0490**	0.47554
	NTKDTNVWKGDY	129-140	HLA-A*30:02 HLA-A*01:01	**1.0338**	0.07828
	ERFNISVGQIYY	570-581	HLA-A*30:02 HLA-A*01:01 HLA-A*29:02	**0.6841**	0.07431
	LGSVPIFYSPYL	205-216	HLA-B*51:01	**0.7025**	0.04979
	GQPQPVRTVDAL	96-107	HLA-B*07:02	**0.9072**	0.07788
	TESRTGADNINW	583-594	HLA-B*44:02 HLA-B*44:03	**1.3921**	0.23133

			HLA-B*57:01		
	MQEGYTQTLEPR	497-508	HLA-B*39:01	**1.3612**	0.09338
	DMQEGYTQTLEP	496-507	HLA-B*39:01	**1.0761**	0.10323
	NQVTTGVTSRVY	553-564	HLA-A*30:02 HLA-A*23:01	**1.0571**	0.11333
	EQVAEIWNARFK	193-204	HLA-A*32:01 HLA-A*25:01	**0.6631**	0.63818
	TYWRISDDWGLR	609-620	HLA-A*31:01	**1.8667**	0.36631
	WMFYWNHNAVID	311-322	HLA-C*07:02 HLA-B*39:01 HLA-B*48:01	**1.3380**	0.41831
	APNFDATITPHY	252-263	HLA-A*29:02	**1.1750**	0.38888
	TKDTNVWKGDYQ	130-141	HLA-A*30:02	**0.7552**	0.13432
	KDTNVWKGDYQM	131-142	HLA-A*30:02	**0.5903**	0.03507
	AEIWNARFKLGS	196-207	HLA-B*40:01	**1.1163**	0.26973
	CYAIRLGYERKL	721-732	HLA-C*06:02	**1.0312**	0.22755
	SRRWMFYWNHNA	308-319	HLA-B*27:05	**0.9188**	0.41222
	MKQRGENRYTIL	154-165	HLA-B*08:01	**0.7567**	0.29612
	EFRYLTQAGAGL	275-286	HLA-B*27:05	**0.5342**	0.0501
**Outer-membrane lipoprotein carrier protein**	KTLWFYNPFVEQ	85-96	HLA-A*32:01 HLA-A*02:01	**0.5371**	0.51613
	KTLWFYNPFVEQ	85-96	HLA-A*02:01 HLA-A*32:01	**0.5371**	0.51613
	KRPNLFNWHMTQ	63-74	HLA-B*27:05	**0.8777**	0.20742

Table S2: Final MHC-II epitopes selected on the basis of different parameters

protein	SN	HTL epitopes	Position	alleles	Antigenicity	IFN-Y	IL-4	IL-10	
**LPS-assembly protein LptD**	1	EFKIDGKLVFERDMQ	484-498	**HLA-DRB1*03:09**<br>HLA-DRB1*03:05<br>HLA-DRB1*03:06<br>HLA-DRB1*03:07<br>HLA-DRB1*03:08<br>HLA-DRB3*01:01<br>HLA-DRB1*11:28<br>HLA-DRB1*13:05<br>HLA-DRB1*03:01<br>HLA-DRB1*11:07	**0.8281**	positive	IL4-inducer	negative	
	2	FKIDGKLVFERDMQE	485-499	**HLA-DRB1*03:09**<br>HLA-DRB1*03:05<br>HLA-DRB1*03:06<br>HLA-DRB1*03:07<br>HLA-DRB1*03:08<br>HLA-DRB1*11:28<br>HLA-DRB1*13:05<br>HLA-DRB1*03:01<br>HLA-DRB1*11:07	**0.6149**	positive	IL4-inducer	negative	
	3	MPEFKIDGKLVFERD	482-496	**HLA-DRB1*03:09**<br>HLA-DRB1*03:05<br>HLA-DRB1*03:01<br>HLA-DRB3*01:01<br>HLA-DRB1*03:06<br>HLA-DRB1*03:07<br>HLA-DRB1*03:08<br>HLA-DRB1*11:28<br>HLA-DRB1*13:05<br>HLA-DRB1*11:07	**0.8419**	positive	IL4-inducer	negative	
	4	PEFKIDGKLVFERDM	483-497	**HLA-DRB1*03:09**<br>HLA-DRB1*03:05<br>HLA-DRB1*03:01<br>HLA-DRB1*03:06<br>HLA-DRB1*03:07<br>HLA-DRB1*03:08<br>HLA-DRB1*11:28<br>HLA-DRB1*13:05<br>HLA-DRB1*11:07	**0.9915**	Positive	IL4-inducer	negative	
	5	VMPEFKIDGKLVFER	481-495	**HLA-DRB1*03:09**<br>HLA-DRB1*03:05<br>HLA-DRB1*03:01<br>HLA-DRB3*01:01<br>HLA-DRB1*03:06<br>HLA-DRB1*03:07<br>HLA-DRB1*03:08<br>HLA-DRB1*11:28	**0.8886**	Positive	IL4-inducer	negative	

				HLA-DRB1*13:05<br>HLA-DRB1*11:07					
	6	QLNYRYASPEYIQAT	650-664	**HLA-DRB1*09:01**<br>HLA-DRB1*07:03	**1.0890**	Positive	IL4-inducer	negative	
	7	VQLNYRYASPEYIQA	649-663	**HLA-DRB1*09:01**<br>HLA-DRB1*07:03<br>HLA-DRB1*11:02<br>HLA-DRB1*11:21<br>HLA-DRB1*13:22	**1.1174**	Positive	IL4-inducer	negative	
	8	LNYRYASPEYIQATL	651-665	**HLA-DRB1*09:01**<br>HLA-DRB1*07:03	**0.8852**	Positive	IL4-inducer	negative	
	15	HLYAQAVHFVNTNGD	409-423	**HLA-DRB1*04:10**	**0.8088**	Positive	IL4-inducer	negative	
	16	LYAQAVHFVNTNGDM	410-424	**HLA-DRB1*04:10**	**0.5398**	Positive	IL4-inducer	negative	
	17	DYQMVGRQGRGTADL	139-153	**HLA-DRB5*01:05**	**1.0729**	Positive	IL4-inducer	negative	
	18	VWKGDYQMVGRQGRG	135-149	**HLA-DRB5*01:05**<br>HLA-DRB1*03:09	**0.7250**	Positive	IL4-inducer	negative	
	19	WKGDYQMVGRQGRGT	136-150	**HLA-DRB5*01:05**<br>HLA-DRB1*03:09	**1.0353**	Positive	IL4-inducer	negative	
	20	QVGMTASWPIVDRWS	680-694	**HLA-DRB1*07:03**	**0.5576**	Positive	IL4-inducer	negative	
	21	SQVGMTASWPIVDRW	679-693	**LA-DRB1*07:03**<br>HLA-DRB1*07:01	**0.5982**	Positive	IL4-inducer	negative	
	22	AGDTYWRISDDWGLR	606-620	**HLA-DRB1*07:03**	**1.6072**	Positive	IL4-inducer	negative	
	23	DTYWRISDDWGLRGG	608-622	**HLA-DRB1*07:03**	**1.4903**	Positive	IL4-inducer	negative	
	24	GDTYWRISDDWGLRG	607-621	**HLA-DRB1*07:03**	**1.5175**	Positive	IL4-inducer	negative	
	25	TYWRISDDWGLRGGI	609-623	**HLA-DRB1*07:03**	**1.3712**	Positive	IL4-inducer	negative	

	26	WAGDTYWRISDDWGL	605-619	**HLA-DRB1*07:03**	**0.7040**	Positive	IL4-inducer	negative	
	27	WRISDDWGLRGGIQY	611-625	**HLA-DRB1*07:03**	**1.6106**	Positive	IL4-inducer	negative	
	28	YWRISDDWGLRGGIQ	610-624	**HLA-DRB1*07:03**	**1.7107**	Positive	IL4-inducer	negative	
	29	GISQVGMTASWPIVD	677-691	**HLA-DRB1*07:01** HLA-DRB1*07:03	**0.6028**	Positive	IL4-inducer	negative	
	30	KEGISQVGMTASWPI	675-689	**HLA-DRB1*07:01** HLA-DRB1*07:03	**0.8819**	Positive	IL4-inducer	negative	
**Outer-membrane lipoprotein carrier protein**	1	DGKTLWFYNPFVEQA	83-97	HLA-DQA1*01:01/DQB1*05:01 1	**0.5614**	positive	IL4-inducer	Negative	
	2	SDGKTLWFYNPFVEQ	82-96	HLA-DQA1*01:01/DQB1*05:01 1	**0.6977**	positive	IL4-inducer	Negative	
	3	VSDGKTLWFYNPFVE	81-95	HLA-DQA1*01:01/DQB1*05:01 1	**0.5433**	positive			

Table S3: Final B Cell epitopes selected on the basis of different parameters

B cell epitopes					
Protein	B cell Epitope	Position	Score	Antigenicity	Immunogenicity
**Lipopolysaccharide export system protein LptA**	TQGTIKINADKVVV	61	0.85	0.6236	0.00862
	LSLNIALASALLAA	8	0.58	1.0434	0.06495
	KMHYELQNDFVVLT	111	0.52	0.9433	0.1772
**LPS-assembly protein LptD**	DNINWENNDTTGSL	590	0.93	0.8324	0.36517
	VMPEFKIDGKLVFE	481	0.91	1.1220	0.06815
	YYWNIAPNFDATIT	247	0.85	1.7077	0.49894
	GYVNQHFDATVSTK	362	0.81	0.8990	0.05901
	SRRWMFYWNHNAVI	308	0.79	0.5648	0.47104
	KLVFERDMQEGYTQ	490	0.77	0.6035	0.16586
	MVGRQGRGTADLMK	142	0.72	0.7063	0.0515
	NSYGAEPQLDINAY	386	0.68	1.7424	0.19838
	AVIDQVWRLNADYT	319	0.66	0.5772	0.29531
	PDNAVFTGNVDINQ	64	0.62	0.5371	0.42559
	STAQQYKEGISQVG	669	0.52	0.6477	-0.39953
**Outer-membrane lipoprotein carrier protein**	LWVKRPNLFNWHMT	60	0.68	1.1223	0.05981
